# Naringin Protects against Tau Hyperphosphorylation in A*β*_25–35_-Injured PC12 Cells through Modulation of ER, PI3K/AKT, and GSK-3*β* Signaling Pathways

**DOI:** 10.1155/2023/1857330

**Published:** 2023-02-15

**Authors:** Qi Qiu, Xia Lei, Yueying Wang, Hui Xiong, Yanming Xu, Huifeng Sun, Hongdan Xu, Ning Zhang

**Affiliations:** ^1^College of Pharmacy, Heilongjiang University of Chinese Medicine, Harbin, Heilongjiang 150040, China; ^2^College of Jiamusi, Heilongjiang University of Chinese Medicine, Jiamusi, Heilongjiang 154007, China; ^3^Hospital of Traditional Chinese Medicine, Wuxi, Jiangsu 214071, China; ^4^Department of Pharmacy, Higher Health Vocational Technology School, Wuxi 214000, China; ^5^Chinese Medicine & Postdoctoral Mobile Research Station, Heilongjiang University of Chinese Medicine, Harbin 150040, China

## Abstract

Alzheimer's disease (AD) is the most common form of dementia and a significant social and economic burden. Estrogens can exert neuroprotective effects and may contribute to the prevention, attenuation, or even delay in the onset of AD; however, long-term estrogen therapy is associated with harmful side effects. Thus, estrogen alternatives are of interest for countering AD. Naringin, a phytoestrogen, is a key active ingredient in the traditional Chinese medicine Drynaria. Naringin is known to protect against nerve injury induced by amyloid beta-protein (A*β*) _25–35_, but the underlying mechanisms of this protection are unclear. To investigate the mechanisms of naringin neuroprotection, we observed the protective effect on A*β*_25–35_-injured C57BL/6J mice's learning and memory ability and hippocampal neurons. Then, an A*β*_25–35_ injury model was established with adrenal phaeochromocytoma (PC12) cells. We examined the effect of naringin treatment on A*β*_25–35_-injured PC12 cells and its relationship with estrogen receptor (ER), phosphatidylinositol 3-kinase/protein kinase B (PI3K/AKT), and glycogen synthase kinase (GSK)-3*β* signaling pathways. Estradiol (E_2_) was used as a positive control for neuroprotection. Naringin treatment resulted in improved learning and memory ability, the morphology of hippocampal neurons, increased cell viability, and reduced apoptosis. We next examined the expression of ER*β*, p-AKT (Ser473, Thr308), AKT, p-GSK-3*β* (Ser9), GSK-3*β*, p-Tau (Thr231, Ser396), and Tau in PC12 cells treated with A*β*_25–35_ and either naringin or E_2_, with and without inhibitors of the ER, PI3K/AKT, and GSK-3*β* pathways. Our results demonstrated that naringin inhibits A*β*_25–35_-induced Tau hyperphosphorylation by modulating the ER, PI3K/AKT, and GSK-3*β* signaling pathways. Furthermore, the neuroprotective effects of naringin were comparable to those of E_2_ in all treatment groups. Thus, our results have furthered our understanding of naringin's neuroprotective mechanisms and indicate that naringin may comprise a viable alternative to estrogen therapy.

## 1. Introduction

Alzheimer's disease (AD), a neurodegenerative disorder characterized by progressive cognitive impairment, is the leading cause of dementia [1]. Recent research has shown that estrogen not only regulates the growth and development of the reproductive system but also serves a neuroprotective role in the pathophysiology of AD [2]. Estrogen receptor *β* (ER*β*) is highly expressed in the hippocampus. Binding of estrogen to ER*β* activates the phosphatidylinositol 3-kinase/protein kinase B (PI3K/AKT) pathway and reduces the toxic effects of amyloid beta-protein (A*β*) on adrenal phaeochromocytoma (PC12) cells [3]. Estrogen also helps repair the central nervous system (CNS), stimulating axon regeneration following subcortical axon injury by modulating the PI3K/AKT pathway [4]. The PI3K/AKT signaling pathway is involved in growth, development, learning, and memory aspects of the CNS, and plays a significant role in the prevention and treatment of AD [5]. Activation of the PI3K/AKT signaling pathway inhibits the activity of glycogen synthase kinase (GSK)-3*β*, which is closely linked to abnormal Tau protein phosphorylation [6]. As the protein kinase that regulates Tau protein phosphorylation, GSK-3*β* is a key factor in the occurrence and development of AD.

Deactivation of GSK-3*β* could potentially retard the development of AD hallmarks namely, A*β* plaques and neurofibrillary tangles (NFTs) [7]. In a dementia mouse model, estrogen deficiency was shown to aggravate cognitive impairment through the ER*β*/GSK-3*β* pathway [8]. Thus, the neuroprotective effects of estrogen in AD are likely related to the ER, PI3K/AKT, and GSK-3*β* signaling pathways.

Unfortunately, long-term estrogen therapy can cause catastrophic side effects such as breast and uterine cancer. Thus, alternatives to estrogen should be investigated as viable candidates for countering AD.

Phytoestrogens are a class of plant-derived compounds similar to estradiol (E_2_) in structure and function. Phytoestrogens bind ER and can impart both estrogen-like and anti-estrogen-like effects without the harmful side effects associated with long-term estrogen therapy [9]. Hence, phytoestrogens are considered viable candidates for the prevention and treatment of AD [10]. Previous research conducted by our group showed that *Drynaria*, a plant used in traditional Chinese medicine, had a significant protective effect on the functionality of the CNS [11]. Naringin, a key active ingredient of *Drynaria fortune*, is a flavonoid phytoestrogen with antioxidant, anti-inflammatory, and anti-apoptotic activities [12]. The estrogen-like activity of naringin has been confirmed [13], making it a viable therapeutic candidate for AD studies. In a recent study, naringin exhibited Mas receptor-mediated neuroprotective effects against A*β*-induced cognitive impairment and mitochondrial toxicity in rats [14]. Another study showed that the nitric oxide signaling pathway is involved in naringin's regulation of intranasal manganese and intraventricular A*β*-induced neurotoxicity [15]. However, studies examining the effect of naringin on the ER, PI3K/AKT, and GSK-3*β* signaling pathways are lacking.

In the present study, we investigated the protective effects of naringin on A*β*_25–35_-injured C57BL/6J mice and A*β*_25–35_-injured PC12 cells, and compared them to the effects of E_2_. In cell experiments, the ER inhibitor ICI182780, the PI3K inhibitor LY294002, and the GSK-3*β* inhibitor LiCl were used to study the relationship between naringin's protective effects and the ER, PI3K/AKT, and GSK-3*β* signaling pathways. We found the neuroprotective effects of naringin were comparable to those of E_2_ and involve the inhibition of A*β*_25–35_-induced Tau phosphorylation through modulation of the ER, PI3K/AKT, and GSK-3*β* signaling pathways.

## 2. Materials and Methods

### 2.1. Animals

Eight-week-old male C57BL/6J mice were purchased from Beijing Vital River Laboratory Animal Technology Co., Ltd. (Beijing, China). Mice were raised in specific pathogen free (SPF)-grade animal facilities, with a temperature of 20–22°C and relative humidity of 55–65%. The light source was natural, and the light and dark alternated for 12 hours (12L : 12D), with ad libitum access to water and food. In order to make the mice adapt to the environment, they were adaptively fed for 1 week before the experiment. The operations were carried out under general anesthesia to reduce the pain of mice. The study was approved by the Ethical Committee of Heilongjiang University of Chinese Medicine (2020092502) and was conducted according to accepted animal care practices.

### 2.2. Cell Culture

PC12 cells purchased from Shanghai Zhong Qiao Xin Zhou Biotechnology Co., Ltd. (Shanghai, China) were cultured in standard dulbecco's modified eagle medium (DMEM) supplemented with 10% fetal bovine serum (FBS) at 37°C and 5% CO_2_.

### 2.3. Treatment Reagents

A*β*_25–35_ was purchased from Beijing Boosen Biological Technology Co., Ltd. (Beijing, China). E_2_ and naringin were purchased from J&K Scientific Ltd. (Beijing, China). Dye for hematoxylin eosin (H&E) staining was purchased from Beyotime Biotechnology (Shanghai, China). ICI182780 and LY294002 were purchased from Selleck Chemicals LLC (Houston, TX, USA). LiCl was purchased from Sigma-Aldrich (St. Louis, MO, USA).

### 2.4. Experimental Treatments

**Figure 1:**
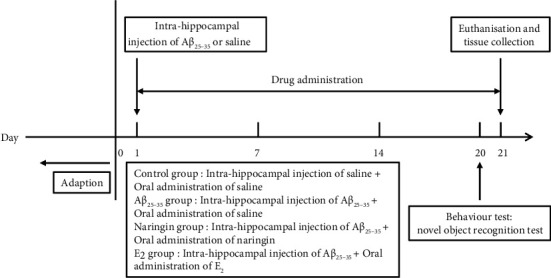
Timeline of animal experimental procedures.

C57BL/6J mice were subjected to the following treatments: 24 mice were randomly assigned into the control group, A*β*_25–35_ group, naringin group, and E_2_ group, with 6 mice in each group. The A*β*_25–35_ group, naringin group, and E_2_ group were anesthetized by inhaling isoflurane with portable small animal anesthesia machine (ZS-MV-IV, Beijing Zhongshidichuang Science and Technology Development Co., Ltd., Beijing, China) and fixed on the stereotaxic apparatus (ZS-FD/S, Beijing Zhongshidichuang Science and Technology Development Co., Ltd., Beijing, China). The mouse's scalp was cut open to expose the skull. At the injection point in the hippocampal regions (2.0 mm behind the Bregma, 2.1 mm from the lateral to the left and right, 3.7 mm deep under the skull surface), holes were drilled into the surface with a skull drill. A*β*_25–35_ 1 *μ*l (10 *μ*g/*μ*l) was subsequently injected on each side with a microinjection needle. A*β*_25–35_ 1 *μ*l was injected over the course of 5 minutes, and the needle was kept in place for an additional 10 minutes after the injection was completed. The scalp was then sutured closed. Normal saline, naringin 100 mg kg^−1^ d^−1^, or E_2_ 0.13 mg kg^−1^ d^−1^ was administered according to the group classification for 21 days, with an oral administration at the dosage of 10 mL kg^−1^ d^−1^. After the control group was anesthetized in the same way, the same amount of normal saline was injected into the bilateral hippocampus, and the same amount of normal saline was given by oral administration for 21 days (Figure 1).

Logarithmic phase PC12 cells were subjected to the following treatments: (1) Untreated: DMEM for 24 hours; (2) A*β*_25–35_: 20 *μ*M A*β*_25–35_ for 24 hours; (3) E_2_:100 pM or 1 nM E_2_ for 24 hours; (4) naringin: 100 pM, 1 nM, 10 nM, 100 nM, 1 M, 10 M, 100 M, or 1 mM naringin for 24 hours; (5) E_2_ + A*β*_25–35_: 1 nM E_2_ for 2 hours, followed by 20 *μ*M A*β*_25–35_ for 24 hours; (6) naringin + A*β*_25–35_: 100 pM, 1 nM, 10 nM, 100 nM, 1 M, 10 M, 100 M, or 1 mM naringin for 2 hours, followed 20 *μ*M A*β*_25–35_ for 24 hours; (7) ICI182780 + E_2_:1 M ICI182780 for 1 hour, followed by 100 pM E_2_ for 24 hours; (8) ICI182780 + naringin: 1 M ICI182780 for 1 hour, followed by 1 mM naringin for 24 hours; (9) ICI182780 + E_2_ + A*β*_25–35_: 1 M ICI182780 for 1 hour, followed 1 nM E_2_ for 2 hours, followed by 20 *μ*M A*β*_25–35_ for 24 hours; (10) ICI182780 + naringin + A*β*_25–35_: 1 M ICI182780 for 1 hour, followed by 1 M naringin for 2 hours, followed by 20 *μ*M A*β*_25–35_ for 24 hours; (11) LY294002 + E_2_ + A*β*_25–35_: 50 *μ*M LY294002 for 1 hour, followed by 1 nM E_2_ for 2 hours, followed by 20 *μ*M A*β*_25–35_ for 24 hours; (12) LY294002 + naringin + A*β*_25–35_: 50 *μ*M LY294002 for 1 hour, followed by 1 M naringin for 2 hours, followed by 20 *μ*M A*β*_25–35_ for 24 hours; (13) LiCl + A*β*_25–35_: 10 mM LiCl for 2 hours, followed by 20 *μ*M A*β*_25–35_ for 24 hours.

### 2.5. Novel Object Recognition Test

To examine learning and memory, we performed the novel object recognition (NOR) test [16]. The test is based on animals' innate preference for a novel object, in which mice remembering the familiar object will devote more time exploring a novel one [17]. Each mouse was placed in a box (without objects) for 5 minutes to adapt to the surroundings (habituation). Two identical small cubes were placed at adjacent corners in the box. Each mouse was positioned with its back to the cubes and placed at the midpoint of the opposite box wall. Then, each mouse was given 5 minutes to explore the new objects (training). One hour after the training period, one of the small cubes was replaced with a completely different novel object (Novel Object Recognition Equipment, Shanghai Yishu Information Technology Co., Ltd., Shanghai, China). Each mouse was placed at the same position in the box, and the time taken by each mouse to explore the novel object (Tn) and to explore the familiar object (Tf) within 5 minutes was recorded (test). Objects and the arena were thoroughly cleaned with 70% ethanol between experiments with individual mice to ensure that their behavior was not guided by odor cues [18]. We used the following indices: Discrimination index = (Tn − Tf)/(Tn + Tf). Recognition index = Tn/(Tn + Tf).

### 2.6. H&E Staining

After the NOR test, the mice were euthanized per protocol and their brains were removed, the residual blood was washed with normal saline, and the brains were fixed with 4% paraformaldehyde for 48 hours, and stained pathological sections were prepared. The brain tissue fixed in 4% paraformaldehyde was dehydrated and embedded by conventional pathological methods to make wax blocks, and we used a rotary slicer to cut the wax block into brain sections (4.0 *μ*m thick), dewaxed in xylene, and dehydrated with 95% ethanol. Then, the sections were stained with H&E solution and dehydrated with graded alcohol. We then made the sections transparent with xylene and sealed the sections with neutral glue. H&E staining was performed to observe the morphology of the hippocampus.

### 2.7. MTT Cell Viability Assay

Following treatment, cells were collected into 96-well plates. Twenty microliters of a 5 g/L methyl thiazolyl tetrazolium (MTT) solution (Sigma-Aldrich) were added to each well, and the plates were incubated at 37°C for 4 hours. Following removal of the supernatant, 150 L of dimethylsulfoxide (DMSO) solution was added to each well. Plates were incubated at 37°C for 10 minutes on a shaker, and the absorbance was measured at 570 nm on a microplate reader (MK3; Shanghai Thermoelectric Instrument Co., Ltd., Shanghai, China). The percentage of normalized cell proliferation was calculated as follows: absorbance value of treated cells/absorbance value of untreated cells × 100%.

### 2.8. Annexin V-FITC/PI Double Staining Flow Cytometry Apoptosis Assay

Treated cells were collected into 6-well plates; digested with trypsin; washed twice with PBS; and incubated in 195 *μ*L Annexin V-FITC binding solution, 5 *μ*L Annexin V-FITC, and 10 *μ*L PI staining solution at room temperature in the dark for 15 minutes. During the incubation, a pipette gun was blown every 5 minutes to improve the dyeing effect. Processed cells were analyzed by flow cytometry within 1 hour, and the results were processed using the FlowJo flow cytometry software package (BD Biosciences, Ashland, OR, USA).

### 2.9. qRT-PCR

Treated cells were collected into 6-well plates. Total RNA was extracted, and its quality was detected. CDNA was synthesized and amplified using the Ultra SYBR Mixture kit (Biosharp Life Sciences, Seoul, Korea) on a MX3000p fluorescent quantitative PCR instrument (Agilent Technologies, Santa Clara, CA, USA). The PCR primer sequences were: (1) ER*α* forward: 5′-ATGGGCACTTCAGGAGACAT-3′ and reverse: 5′-AAAGGTGGTTCAGCATCCAA-3′; (2) ER*β* forward: 5′-TCTGGGTGATTGCGAAGAG-3′ and reverse: 5′-TGCCCTTGTTACTGATGTGC-3′; (3) *β*-actin forward: 5′-TGTCACCAACTGGGACGATA-3′ and reverse: 5′-GGGGTGTTGAAGGTCTCAAA-3′. The PCR cycling parameters were: 1 cycle of denaturation at 95°C for 10 minutes; followed by 40 cycles of denaturation at 95°C for 30 seconds, annealing at 60°C/57°C for 1 minute, and extension at 74°C for 1 minute. CT values, dissolution curves, and amplification curves were calculated by the PCR instrument. The relative expressions of ER*α* and ER*β* were calculated by the 2^−∆∆Ct^ method.

### 2.10. Immunoblot Assays

Treated cells were collected into 6-well plates and lysed with radioimmunoprecipitation assay buffer (RIPA) buffer containing 1 mM phenylmethanesulfonyl fluoride (PMSF) for 30 minutes on ice. Following total protein extraction, protein concentration was determined using the bicinchoninic acid assay (BCA) method. Proteins were separated by SDS-polyacrylamide gel electrophoresis (PAGE) and transferred to polyvinylidene fluoride (PVDF) membranes. The PVDF membranes were incubated overnight at 4°C with the following primary antibodies: rabbit anti-ER*β*, rabbit anti-p-AKT (Ser473, Thr308), rabbit anti-AKT, rabbit anti-p-GSK-3*β* (Ser9), rabbit anti-p-Tau (Thr231, Ser396), rabbit anti-Tau (all purchased from Beijing Boosen Biological Technology Co., Ltd., China), and mouse anti-GSK-3*β* (Wuhan Boster Biological Technology Co., Ltd., Wuhan, China). Membranes were washed and incubated with horseradish peroxidase (HRP)-labeled goat anti-mouse IgG or HRP-labeled goat anti-rabbit IgG (Wuhan Boster Biological Technology Co., Ltd.) at room temperature for 1 hour. Membranes were washed again, the enhanced chemiluminescence (ECL) kit (Beyotime Biotechnology) was used to detect immunoreactive bands. A lane ID gel analysis system was used to analyze the grey values.

### 2.11. Statistical Analyses

All statistical analyses were performed using SPSS version 21.0 (IBM Corp., Armonk, NY, USA). One-way analysis of variance (ANOVA) was implemented for comparison between groups, least significant difference (LSD) tests were applied to compare means between groups, and SNK was used for post-statistical corrections. Experimental data were expressed as mean ± standard deviation ($\bar{x\;}\pm S$), and statistical significance was set at *P* < 0.05.

## 3. Results

### 3.1. Effect of Naringin on Learning and Memory Ability of C57BL/6J Mice Injured by A*β*_25–35_

**Figure 2:**
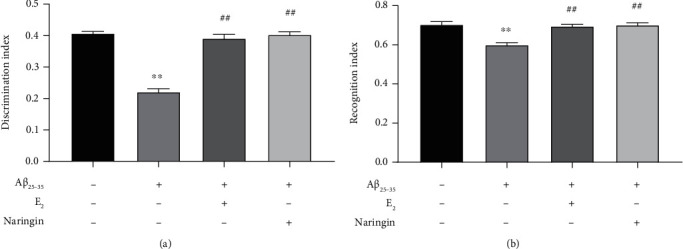
Effect of naringin on learning and memory ability of C57BL/6J mice injured by A*β*_25–35_. The learning and memory ability of mice in each group was tested by a novel object recognition test. (a) Discrimination index in novel object recognition test. (b) Recognition index in a novel object recognition test. Data are expressed as mean ± SD (*n* = 6). Compared with the control group, ∗∗*P* < 0.01; compared with the A*β*_25–35_ group, ^##^*P* < 0.01.

After A*β*_25–35_ treatment, compared with the control group, the discrimination index of A*β*_25–35_-injured C57BL/6J mice significantly decreased. In contrast, in the naringin group, the discrimination index significantly increased, which reflects the ability of learning and memory improvement. Additionally, the amount of time that A*β*_25–35_-injured mice spent exploring the novel object was significantly less than that of the control group, as these mice were less interested in exploring and learning about the novel object. However, the mice supplemented with naringin were observed to have a significant increase in preference for the novel object (Figure 2).

### 3.2. Effect of Naringin on Morphology of Hippocampal Neurons of C57BL/6J Mice Injured by A*β*_25–35_

**Figure 3:**
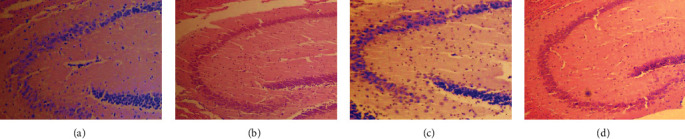
Effect of naringin on morphology of hippocampal neurons of C57BL/6J mice injured by A*β*_25–35_. H&E staining was used to observe the morphology of hippocampal neurons of mice in each group. (a) Representative images of H&E-stained neurons in the control group. (b) Representative images of H&E-stained neurons in the A*β*_25–35_ group. (c) Representative images of the H&E-stained neurons in the naringin group. (d) Representative images of H&E-stained neurons in the E_2_ group.

In the control group, the cells in the hippocampus were arranged regularly, the number of neurons was large, the staining was uniform, the nucleolus was clearly visible, and there was no nuclear shrinkage. After the A*β*_25–35_ treatment, compared with the control group, the hippocampal cells in the model group were disordered, some neurons were lost, the number of neurons was reduced, the cells were reduced, the nucleolus was not obvious, and nuclear shrinkage was obvious. In contrast, the arrangement of hippocampal neurons in the naringin group was more regular, there were more neurons, the morphology was normal, the nucleolus was clear, and there was no obvious nuclear shrinkage (Figure 3).

### 3.3. Effect of Naringin on Viability of PC12 Cells Injured by A*β*_25–35_

**Figure 4:**
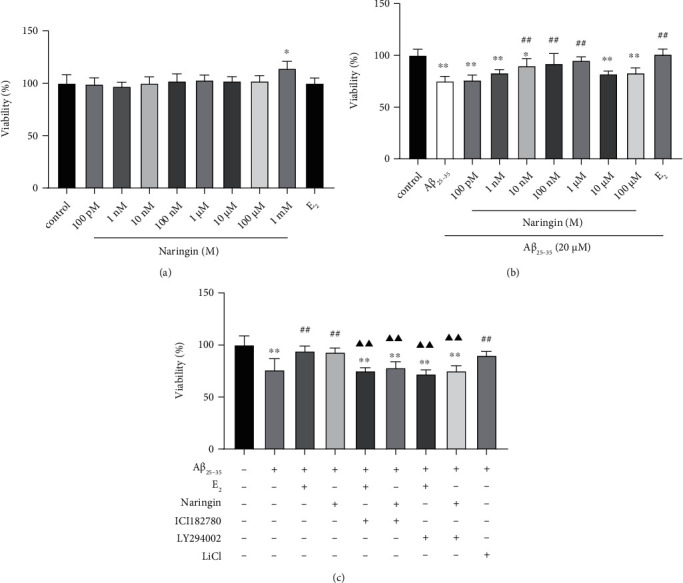
Effect of naringin on the viability of PC12 cells injured by A*β*_25–35_. Cell viability was measured by MTT assays after exposure to the following treatments. (a) PC12 cells were incubated with different concentrations of naringin (100 pM, 1 nM, 10 nM, 100 nM, 1 *μ*M, 10 *μ*M, 100 *μ*M, and 1 mM) or E_2_ (1 nM) for 24 hours. (b) PC12 cells were incubated with naringin (100 pM, 1 nM, 10 nM, 100 nM, 1 *μ*M, 10 *μ*M, and 100 *μ*M) or E_2_ (1 nM) for 2 hours, then treated with A*β*_25–35_ (20 *μ*M) for another 24 hours. (c) PC12 cells were precultured with ICI182780 (1 *μ*M) or LY294002 (50 *μ*M) for 1 hour, then treated with naringin (1 *μ*M) or E_2_ (1 nM) for 2 hours, and then treated with A*β*_25–35_ (20 *μ*M) for another 24 hours; or precultured with LiCl (10 mM) for 2 hours, then treated with A*β*_25–35_ (20 *μ*M) for another 24 hours. Data are expressed as the mean ± SD (*n* = 6). Compared with the untreated cells, ∗*P* < 0.05, ∗∗*P* < 0.01; compared with A*β*_2s5–35_-treated cells, ^##^*P* < 0.01; compared with the treatment group, ^▲▲^*P* < 0.01.

Next, we used PC12 cells to further study the in-depth mechanism of naringin's neuroprotective effect. Previous experiments conducted by our group showed that 1 nM E_2_ had no significant effect on PC12 cell viability and was the optimal concentration to mitigate injury by A*β*_25–35_. Therefore, treatment with 1 nM E_2_ was used as a positive control in experiments assessing the efficacy of naringin. To determine the optimal concentration of naringin, we utilized the MTT method to detect cell viability. First, we determined the effect of different naringin concentrations on the viability of PC12 cells. Relative to untreated cells, the viability of PC12 cells treated with 1 mM naringin was significantly increased (Figure 4(a)). Naringin concentrations ranging from 100 pM to 100 M had no significant effect on PC12 cell viability and were therefore used in subsequent experiments.

We next examined whether naringin pretreatment would protect cells from the effects of A*β*_25–35_. As expected, the viability of PC12 cells treated with 20 M A*β*_25–35_ was significantly lower than that of untreated cells, and the cytotoxic effect of A*β*_25–35_ was abrogated by E_2_ pretreatment (Figure 4(b)). Pretreating cells with naringin also successfully abrogated the effect of A*β*_25–35_ on cell viability, with a naringin concentration of 1 M providing protection similar to that of E_2_.

To investigate the roles of the ER, PI3K/AKT, and GSK-3*β* pathways in A*β*_25–35_-induced cytotoxicity, we examined the effects of the ER blocker ICI182780, the PI3K inhibitor LY294002, and the GSK-3*β* inhibitor LiCl on A*β*_25–35_-induced PC12 cell injury. As previously observed, the cytotoxic effects of A*β*_25–35_ were significantly abrogated by pretreatment with either naringin or E_2_ (Figure 4(c)). Pretreatment with LiCl also significantly safeguarded cells against A*β*_25–35_-induced injury, suggesting that naringin may exert its protective effects, at least in part, by inhibiting GSK-3*β* activity. The protective effects of naringin and E_2_ against A*β*_25–35_-induced PC12 cell injury were lost when cells were first pretreated with either ICI182780 or LY294002. The above data indicate that naringin's protective effects against A*β*_25–35_-induced cell injury may be mediated through activation of the ER and/or PI3K/AKT signaling pathways.

### 3.4. Effect of Naringin on the Rate of Apoptosis in PC12 Cells Injured by A*β*_25–35_

**Figure 5:**
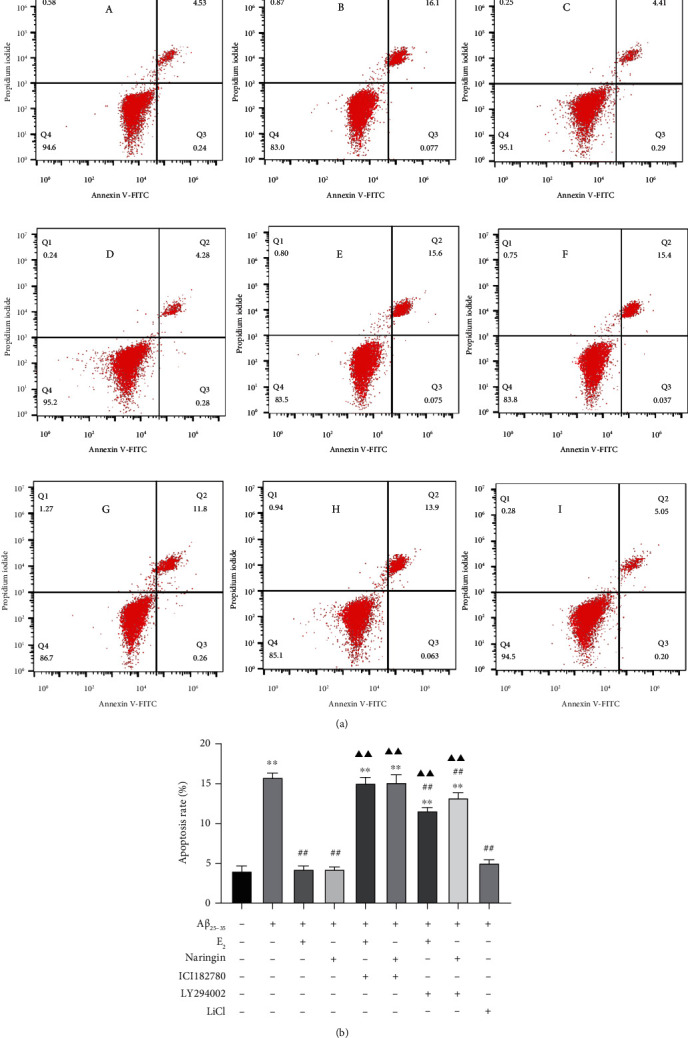
Effect of naringin on the apoptosis rate of PC12 cells injured by A*β*_25–35_. Apoptosis was monitored by Annexin V-FITC/PI double stain flow cytometry after exposure to the following treatments. (a) Panel A, untreated; panel B, A*β*_25–35_ treatment; panel C, E_2_ + A*β*_25–35_ treatment; panel D, naringin + A*β*_25–35_ treatment; panel E, ICI182780 + E_2_ + A*β*_25–35_ treatment; panel F, ICI182780 + naringin + A*β*_25–35_ treatment; panel G, LY294002 + E_2_ + A*β*_25–35_ treatment; panel H, LY294002 + naringin + A*β*_25–35_; panel I, LiCl + A*β*_25–35_ group. (b) Quantitative analysis of apoptosis rates. Data are expressed as the mean ± SD (*n* = 3). Compared with untreated cells, ∗∗*P* < 0.01; compared with A*β*_25–35_-treated cells, ^##^*P* < 0.01; compared with the treatment group, ^▲▲^*P* < 0.01.

We further examined the roles of the ER, PI3K/AKT, and GSK-3*β* pathways in A*β*_25–35_-induced cytotoxicity and naringin neuroprotection by monitoring the rate of apoptosis in different treatment groups. Utilizing Annexin V-fluorescein isothiocyanate/propidium iodide (V-FITC/PI) double stain flow cytometry, we found that the apoptosis rate of A*β*_25–35_-treated PC12 cells was significantly higher than that of untreated cells (Figure 5(a) panels A and B; Figure 5(b)). Pretreatment of cells with naringin, LiCl, or E_2_ prior to A*β*_25–35_ exposure significantly decreased the rate of apoptosis (Figure 5(a) panels C, D, and I; Figure 5(b)). Preexposure to the ER blocker ICI182780 or the PI3K inhibitor LY294002 abrogated the protective effects of naringin and E_2_ against A*β*_25–35_-induced cytotoxicity, resulting in increased rates of apoptosis (Figure 5(a) panels E–H; Figure 5(b)). These results provide further evidence that naringin may protect PC12 cells from A*β*_25–35_-induced injury by inhibiting GSK-3*β* activity and activating the ER and/or PI3K/AKT signaling pathways.

### 3.5. The Role of the ER Pathway in Naringin Protection

**Figure 6:**
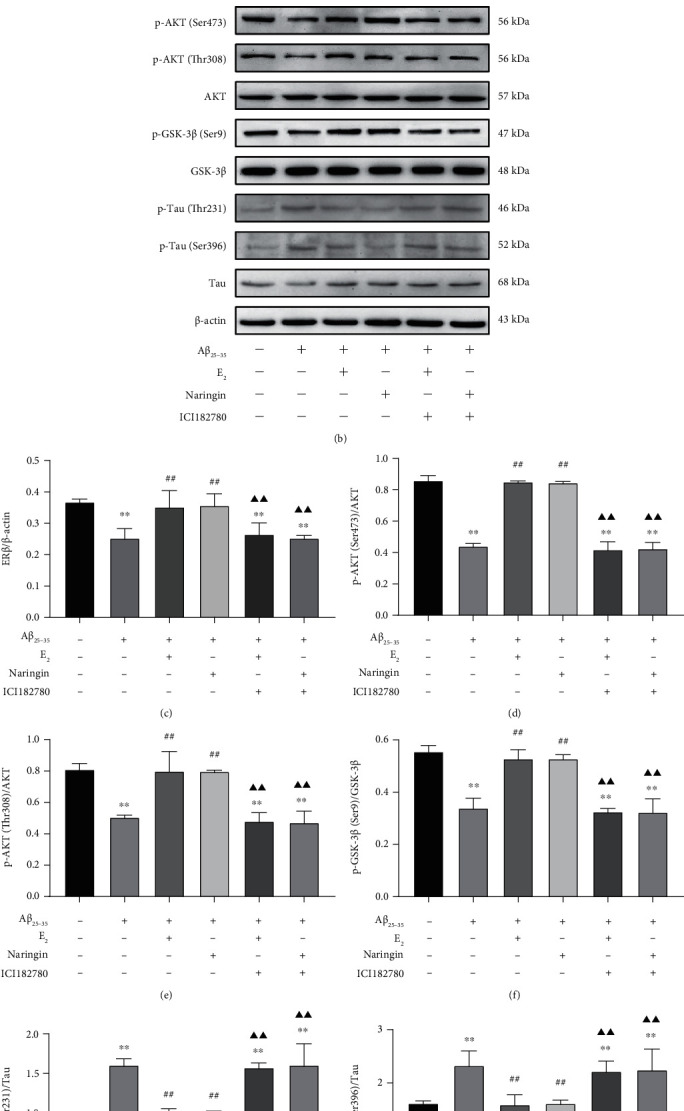
Effect of naringin on the ER pathway in the context of A*β*_25–35_-induced injury. (a) Relative qRT-PCR was implemented to monitor the expression of ER mRNA. (b) Immunoblots were used to assess levels of ER*β*, p-AKT (Ser473, Thr308), AKT, p-GSK-3*β* (Ser9), GSK-3*β*, p-Tau (Thr231, Ser396), and Tau proteins. (c) Quantitative analysis of ER*β*/*β*-actin protein levels. (d) Quantitative analysis of p-AKT (Ser473)/AKT protein levels. (e) Quantitative analysis of p-AKT (Thr308)/AKT protein levels. (f) Quantitative analysis of p-GSK-3*β* (Ser9)/GSK-3*β* protein levels. (g) Quantitative analysis of p-Tau (Thr231)/Tau protein levels. (h) Quantitative analysis of p-Tau (Ser396)/Tau protein levels. Data are expressed as mean ± SD (*n* = 3). Compared with untreated cells, ∗∗ *P* < 0.01; compared with A*β*_25–35_-treated cells, ^##^*P* < 0.01; compared with the treatment group, ^▲▲^*P* < 0.01.

We hypothesized that naringin exerts its protective effects over A*β*_25–35_-induced injury by modulating the PI3K/AKT and GSK-3*β* signaling pathways through activation of the ER pathway. To test this hypothesis, we first monitored the levels of ER subtype mRNAs in treated PC12 cells by qRT-PCR. As we previously found 100 pM E_2_ and 1 mM naringin to be the most effective concentrations in PC12 cell viability assays, we used these concentrations for our ER expression analyses. Reverse-transcribed total RNA from PC12 cells treated with E_2_ or naringin, alone and in combination with ICI182780, served as the template for quantitative real-time polymerase chain reaction (qRT-PCR) reactions with primers specific to ER*α* and ER*β*. As levels of ER*α* mRNA in cells were exceptionally low, it was difficult to quantitatively amplify Er*α*, and we thus instead chose to focus on ER*β*. Compared with untreated cells, the level of ER*β* mRNA was significantly increased in naringin-treated cells (Figure 6(a)). Exposure of PC12 cells to both ICI182780 and naringin significantly reduced ER*β* mRNA levels compared to treatment with naringin alone. Similar results were seen for E_2_ treatment in the presence and absence of ICI182780. These results indicate that ER*β* mRNA expression can be upregulated by naringin.

We next examined levels of ER*β*, p-AKT (Ser473), p-AKT (Thr308), AKT, p-GSK-3*β* (Ser9), GSK-3*β*, p-Tau (Ser396), p-Tau (Thr231), and Tau proteins by quantitative immunoblotting. Levels of ER*β*/-actin, p-AKT (Ser473, Thr308)/AKT, and p-GSK-3*β* (Ser9)/GSK-3*β* were significantly lower in A*β*_25–35_-treated PC12 cells compared to untreated cells (Figures 6(b), 6(c), 6(d), 6(e), and 6(f)), whereas levels of p-Tau (Thr231, Ser396)/Tau were significantly higher in A*β*_25–35_-treated cells compared to untreated cells (Figures 6(b), 6(g), and 6(h)). Compared to treatment with A*β*_25–35_ alone, treatment with naringin + A*β*_25–35_ resulted in significantly increased levels of ER*β*, p-AKT (Ser473, Thr308)/AKT, and p-GSK-3*β* (Ser9)/GSK-3*β* and significantly decreased levels of p-Tau (Thr231, Ser396)/Tau. Addition of the ER blocker ICI182780 to naringin + A*β*_25–35_ treatment reversed the effect of naringin on the above protein levels. The effect of naringin was similar to that of E_2_ with all treatments, as shown in Figures 6(b), 6(c), 6(d), 6(e), 6(f), 6(g), and 6(h). These results show that naringin promotes the expression of ER*β*, the phosphorylation of AKT and GSK-3*β*, and the inhibition of Tau phosphorylation. The effect of ICI182780 on naringin-mediated expression indicates that naringin likely modulates the PI3K/AKT and GSK-3*β* signaling pathways by activating the ER pathway.

### 3.6. The Role of PI3K/AKT Pathway in Naringin Protection

**Figure 7:**
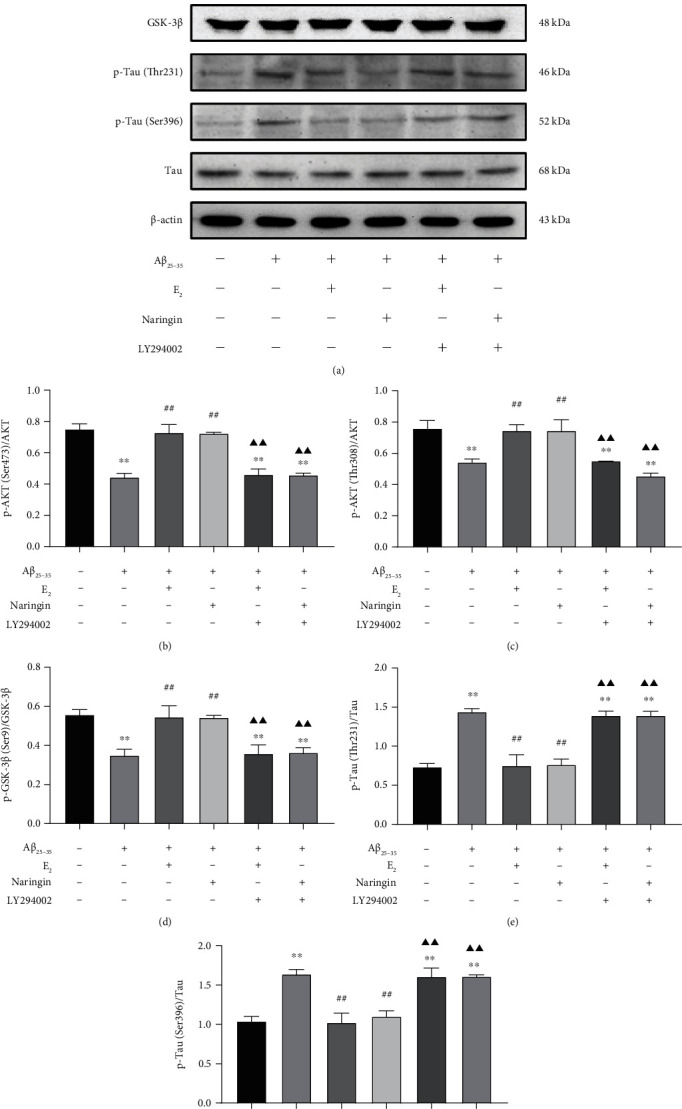
Effect of naringin on the PI3K/AKT pathway in the context of A*β*_25–35_-induced injury. (a) Immunoblots were used to assess the levels of p-AKT (Ser473, Thr308), AKT, p-GSK-3*β* (Ser9), GSK-3*β*, p-Tau (Th231, Ser396), and Tau proteins. (b) Quantitative analysis of p-AKT (Ser473)/AKT protein levels. (c) Quantitative analysis of p-AKT (Thr308)/AKT protein levels. (d) Quantitative analysis of p-GSK-3*β* (ser9)/GSK-3*β* protein levels. (e) Quantitative analysis of p-Tau (Thr231)/Tau protein levels. (f) Quantitative analysis of p-Tau (Ser396)/Tau protein levels. Data are expressed as mean ± SD (*n* = 3). Compared with untreated cells, ∗∗*P* < 0.01; compared with A*β*_25–35_-treated cells, ^##^*P* < 0.01; compared with the treatment group, ^▲▲^*P* < 0.01.

As the PI3K/AKT and GSK-3*β* signaling pathways are highly associated, we wished to further investigate whether naringin modulates GSK-3*β* activation through the PI3K/AKT pathway. We examined the effect of treatment with the PI3K inhibitor LY294002 on levels of p-AKT (Ser473), p-AKT (Thr308), AKT, p-GSK-3*β* (Ser9), GSK-3*β*, p-Tau (Ser396), p-Tau (Thr231), and Tau protein by quantitative immunoblot assays. Compared with untreated PC12 cells, treatment with A*β*_25–35_ alone resulted in significantly decreased levels of p-AKT (Ser473, Thr308)/AKT, and p-GSK-3*β* (Ser9)/GSK-3*β* and significantly increased levels of p-Tau (Thr231, ser396)/Tau (Figure 7). Compared with cells treated with A*β*_25–35_alone, cells treated with A*β*_25–35_ + naringin had significantly increased levels of p-AKT (Ser473, Thr308)/AKT and p-GSK-3*β* (Ser9)/GSK-3*β* and significantly decreased levels of p-Tau (Thr231, Ser396)/Tau. The addition of LY294002 to naringin + A*β*_25–35_ treatment reversed the effect of naringin on the above protein levels. The effect of naringin was similar to that of E_2_ in all treatment groups. These results indicate that naringin promotes GSK-3*β* phosphorylation and inhibits Tau phosphorylation by modulating the PI3K/AKT signaling pathway.

### 3.7. The Role of the GSK-3*β* Pathway in Naringin Protection

**Figure 8:**
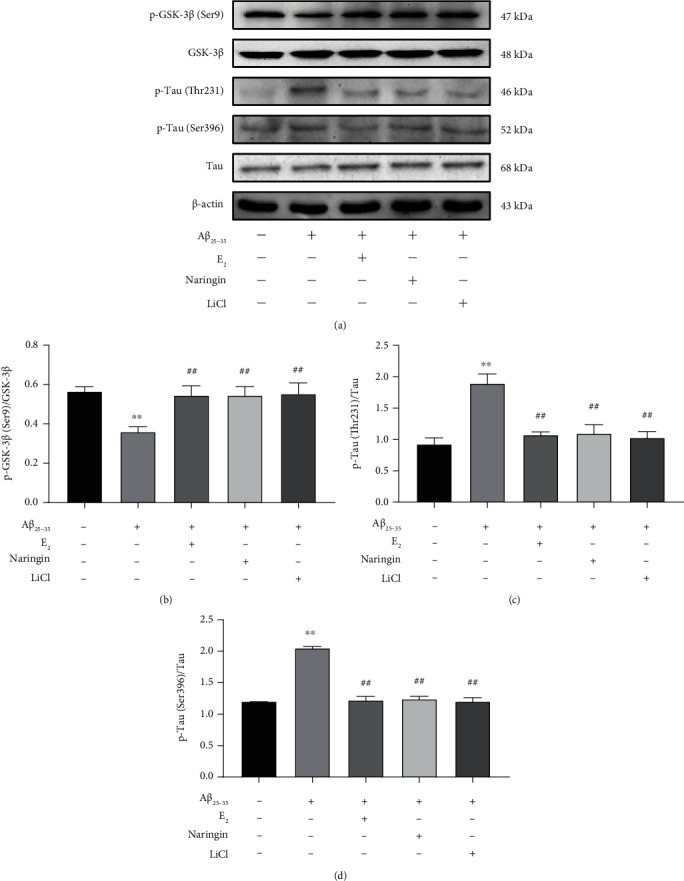
Effect of naringin on the GSK-3*β* pathway in the context of A*β*_25–35_-induced injury. (a) Immunoblots were used to assess the levels of p-GSK-3*β* (Ser9), GSK-3*β*, p-Tau (Thr231, Ser396), and Tau proteins. (b) Quantitative analysis of p-GSK-3*β* (Ser9)/GSK-3*β* protein levels. (c) Quantitative analysis of p-Tau (Thr231)/Tau protein levels. (d) Quantitative analysis of p-Tau (Ser396)/Tau protein levels. Data are expressed as mean ± SD (*n* = 3). Compared with untreated cells, ∗∗*P* < 0.01; compared with A*β*_25–35_-treated cells, ^##^*P* < 0.01.

The above results indicate that the inhibition of GSK-3*β* may be one of the mechanisms by which naringin protects PC12 cells from A*β*_25–35_ injury. To further test this hypothesis, levels of p-GSK-3*β* (Ser9), GSK-3*β*, p-Tau (Ser396), p-Tau (Thr231), and Tau protein were examined by quantitative immunoblotting. Compared with untreated PC12 cells, treatment with A*β*_25–35_ resulted in significantly decreased levels of p-GSK-3*β* (Ser9)/GSK-3*β* and significantly increased levels of p-Tau (Thr231, Ser396)/Tau (Figure 8). Treatment with naringin, LiCl, or E_2_ countered the effects of A*β*_25–35_, resulting in significantly increased levels of p-GSK-3*β* (Ser9)/GSK-3*β* and significantly decreased levels of p-Tau (Thr231, Ser396)/Tau compared to treatment with A*β*_25–35_ alone. These results indicate that inhibition of Tau phosphorylation by naringin may be mediated through the GSK-3*β* signaling pathway, suggesting a mechanism by which naringin protects against A*β*_25–35_-induced neurotoxicity.

## 4. Discussion

Alzheimer's disease, the most common degenerative disease of the CNS, is affected by estrogen levels [19]. Senile plaques (SPs) formed by A*β* and NFTs formed by hyperphosphorylated Tau protein are widely accepted neuropathological markers of AD [20]. Studies have shown that compared with A*β*, the density of NFTs formed by hyperphosphorylated Tau is more strongly correlated with cognitive function [21]. Thus, the occurrence and developmental process of Tau protein hyperphosphorylation can be used as a criterion for judging the course of AD [22]. Tau protein mediates the neurotoxic effects of A*β*, and hyperphosphorylated Tau leads to increased A*β* production [23]. Therefore, Tau protein comprises an important therapeutic target in AD drug development.

Tau protein has more than 45 phosphorylation sites. Tau protein molecules with abnormal phosphorylation are separated from microtubules and eventually form NFTs. The affinity between Tau and microtubules is mainly mediated by threonine/serine (Thr/Ser) phosphorylation, with phosphorylation of Thr231 and Ser396 leading to increased NFT formation [24]. Tau protein phosphorylation is regulated by a variety of protein kinases and protein phosphatases. Among them, GSK-3*β* is the most influential protein kinase in driving AD-like Tau protein hyperphosphorylation. The active form of GSK-3*β* co-localizes with NFTs in the AD brain and phosphorylates Tau at multiple AD-related sites, including Ser396 and Thr231 [25]. Furthermore, activation of GSK-3*β* has been shown to induce Tau hyperphosphorylation and cognitive impairment [26]. Hyperphosphorylated Tau leads to increased A*β* production; in turn, A*β* accumulation can also increase the activity of GSK-3*β* [27].

GSK-3*β* activity can be regulated by the PI3K/AKT signaling pathway [28]. PI3K is localized in the cytoplasm and plays an important role in promoting cell survival. AKT, as a Ser/Thr protein kinase, is an important signal transduction protein at the core of the PI3K signaling pathway. Following PI3K activation, AKT localizes to the cell membrane, which promotes its phosphorylation and subsequent activation [29]. Therefore, the activity of PI3K is typically reflected in the phosphorylation status of AKT. Yang et al. found that activated AKT phosphorylates GSK-3*β* at the Ser9 site, thereby inhibiting GSK-3*β* activity and reducing Tau hyperphosphorylation [30].

ER, a member of the nuclear hormone receptor superfamily, is a classical receptor of estrogens and phytoestrogens. Two subtypes of ER, ER*α* and ER*β*, have differing distributions in reproductive organs, the cardiovascular system, the skeletal system, and the CNS. ER*β* is highly distributed and expressed in the CNS, where it plays an important neuroprotective role [31]. Studies have shown that Er*β* helps regulate learning, memory, and cognitive abilities by mediating the effects of estrogen; also, selective ER*β* receptor agonists have been shown to improve the pathological manifestations and clinical symptoms of AD [32].

Increasing evidence indicates that interactions between the ER and PI3K/AKT signaling pathways may play an important role in neuroprotection. For example, Meng et al. showed that Gypenoside XVII can protect against A*β*-induced neurotoxicity through ER-dependent activation of the PI3K/AKT pathway [33]. Thus, the ER, PI3K/AKT, and GSK-3*β* signaling pathways may comprise key targets for the treatment or prevention of AD.

Our results showed that naringin effectively improved learning, memory ability, and the morphology of hippocampal neurons of C57BL/6J mice injured by A*β*_25–35_, as well as the survival of PC12 cells injured by A*β*_25–35_. In addition, we found that naringin stimulated the expression of ER*β*, activated the PI3K/AKT signaling pathway, inhibited the activity of GSK-3*β*, and inhibited the phosphorylation of Tau protein. Our data indicate that the protective effects of naringin on A*β*_25–35_-induced neurotoxicity are achieved, at least in part, by reducing Tau protein phosphorylation through modulation of the ER, PI3K/AKT, and GSK-3*β* signaling pathways. In our experiments, the effects of naringin were similar to those of E_2_, further indicating that naringin possesses estrogen-like activity and may comprise a viable therapeutic agent for the treatment of AD. As *in vitro* experiments, our results possess inherent limitations, and the underlying mechanisms of naringin neuroprotective effects require further evaluation in animal models. However, our results impart important information regarding modulation of the ER, PI3K/AKT, and GSK-3*β* signaling pathways by naringin, thus providing a theoretical basis for future studies examining naringin's neuroprotective effects and viability as an AD therapeutic agent.

## 5. Conclusions

Our results demonstrated that naringin inhibits A*β*_25–35_-induced Tau hyperphosphorylation by modulating the ER, PI3K/AKT, and GSK-3*β* signaling pathways. Furthermore, the effects of naringin were similar to E_2_. Thus, our results have furthered our understanding of naringin's neuroprotective mechanisms.

## Data Availability

All data and materials generated or analyzed during this study are included in this published article.

## Supplementary Materials

Supplementary Materials Supplement Figure 1: graphical abstract of this article.
