# Supplementary material for: Naringin Protects against Tau Hyperphosphorylation in Aβ25–35-Injured PC12 Cells through Modulation of ER, PI3K/AKT, and GSK-3β Signaling Pathways
Source: Behav Neurol. 2023 Feb 15;2023:1857330. doi: 10.1155/2023/1857330 (PMC9946756; doi:10.1155/2023/1857330)
Supplement: Supplementary Materials — Supplement Figure 1: graphical abstract of this article. [file 1857330.f1.pdf]

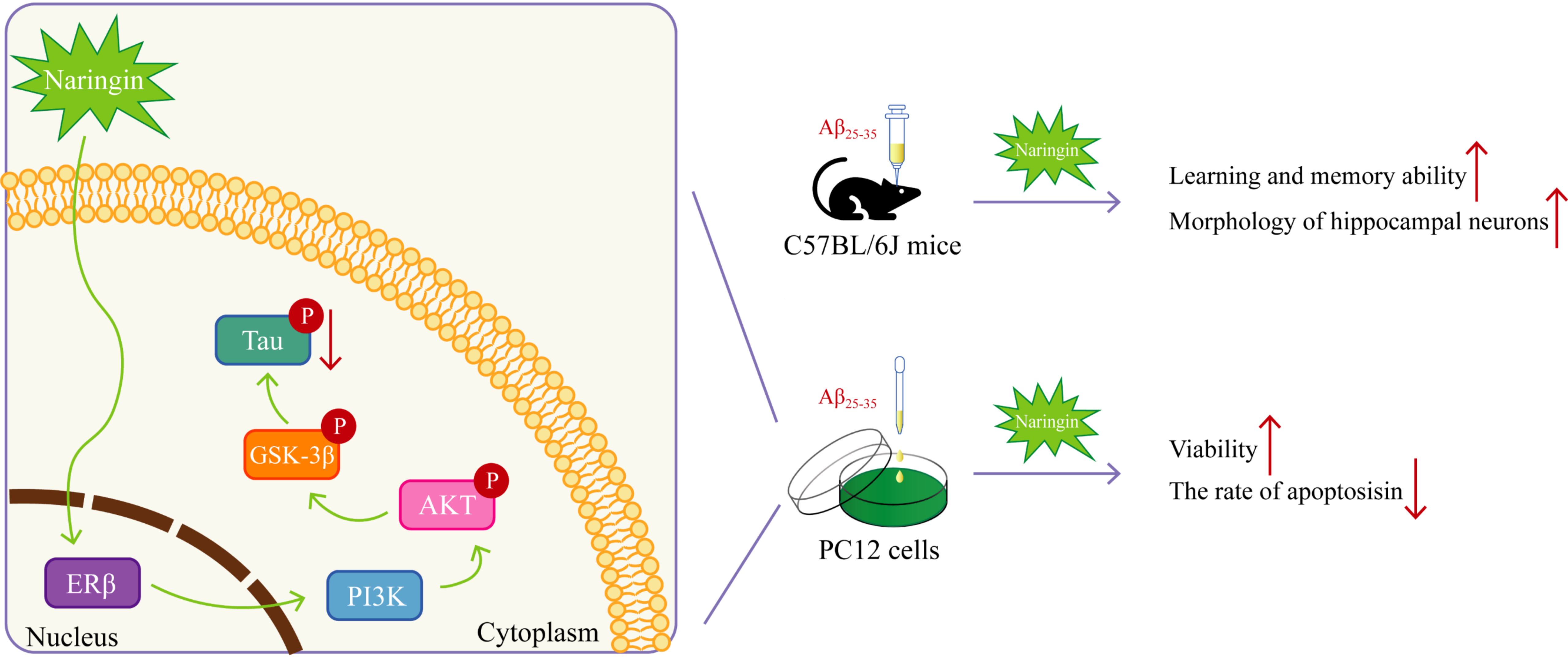
Naringin
Tau
P
GSK-3β
P
AKT
P
ERβ
PI3K
Nucleus
Cytoplasm
Aβ25-35
Naringin
C57BL/6J mice
Learning and memory ability
Morphology of hippocampal neurons
Aβ25-35
Naringin
PC12 cells
Viability
The rate of apoptosisin
